# Granulomatous Interstitial Nephritis Presenting as Hypercalcemia and Nephrolithiasis

**DOI:** 10.1155/2016/4186086

**Published:** 2016-01-19

**Authors:** Saika Sharmeen, Esra Kalkan, Chunhui Yi, Steven D. Smith

**Affiliations:** ^1^Department of Medicine, Mount Sinai St. Luke's-Roosevelt Hospital Center, New York, NY 10025, USA; ^2^Department of Pathology, Mount Sinai St. Luke's-Roosevelt Hospital Center, New York, NY 10025, USA; ^3^Department of Medicine, Division of Nephrology, Mount Sinai St. Luke's-Roosevelt Hospital Center, New York, NY 10025, USA

## Abstract

We report a case of acute kidney injury as the initial manifestation of sarcoidosis. A 55-year-old male was sent from his primary care physician's office with incidental lab findings significant for hypercalcemia and acute kidney injury with past medical history significant for nephrolithiasis. Initial treatment with intravenous hydration did not improve his condition. The renal biopsy subsequently revealed granulomatous interstitial nephritis (GIN). Treatment with the appropriate dose of glucocorticoids improved both the hypercalcemia and renal function. Our case demonstrates that renal limited GIN due to sarcoidosis, although a rare entity, can cause severe acute kidney injury and progressive renal failure unless promptly diagnosed and treated.

## 1. Background

Granulomatous interstitial nephritis (GIN) is a rare cause of acute kidney injury (AKI). Causes of GIN include sarcoidosis, drugs (NSAIDs, antibiotics), and infections (mycobacterial, fungal, bacterial, and viral). Renal involvement as an initial manifestation of sarcoidosis is another rare entity. Renal failure commonly ranges from 0.7% to 4.3% in cases series of patients with previously identified sarcoidosis [1]. The majority of sarcoid related renal failure in these cases is due to two pathologic processes: (1) nephrocalcinosis with or without nephrolithiasis and (2) interstitial nephritis with or without granulomas. We report a case of GIN causing acute kidney injury as the initial presentation of sarcoidosis.

## 2. Clinical Case

**Figure 1:**
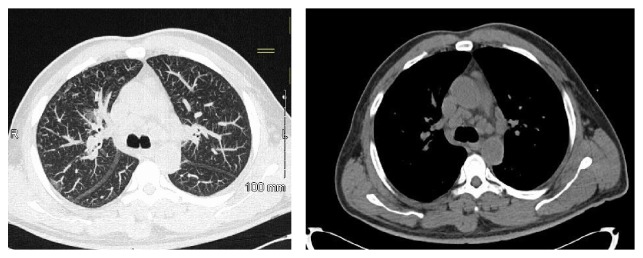
CT chest without IV contrast. Computed tomography without intravenous contrast showing mediastinal and hilar adenopathy.

**Figure 2:**
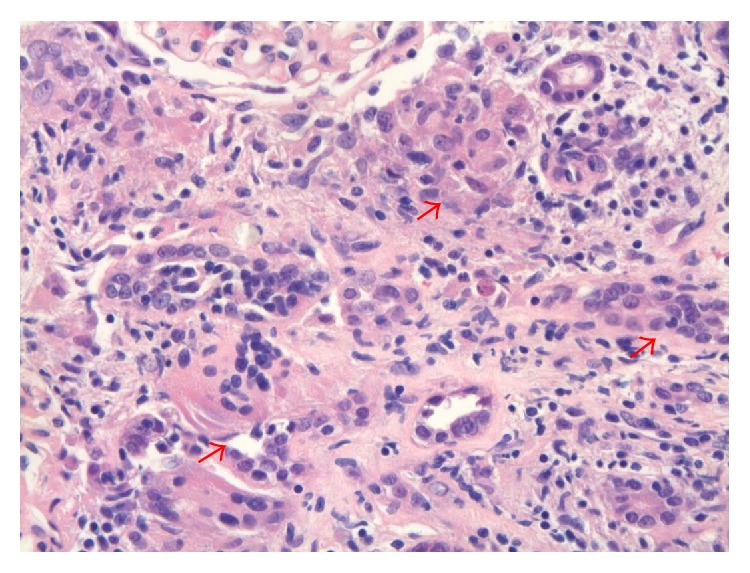
Renal Biopsy. Noncaseating granulomatous inflammation. Aggregation of epithelioid histiocytes aggregation (arrows), mixed with lymphocytes, forming granuloma. Hematoxylin and eosin (HE) stain 400x.

**Figure 3:**
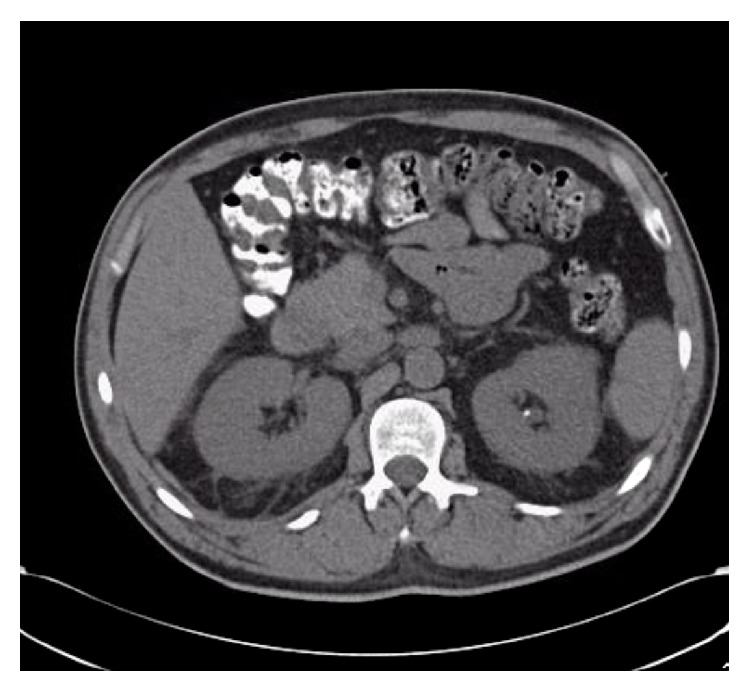
CT of abdomen and pelvis without IV contrast. CT of abdomen and pelvis without IV contrast showing a 3 mm nonobstructing left renal calculus with normal size kidneys and no nephrocalcinosis.

A 55-year-old man was sent from his primary care physician's office with incidental findings of severe hypercalcemia and acute kidney injury (AKI). His medical history was significant for nephrolithiasis and ureteral stone removal one year prior to presentation at which time the serum creatinine was 2.05 mg/dL with a calcium of 10.5 mg/dL. No further work-up was performed at that time. On presentation he was not taking any medications or using alcohol, tobacco, or illicit drugs. He had no prior surgeries. He denied cough, shortness of breath, polyuria, polydipsia, bone pain, and abdominal pain but complained of chronic low back pain and a 20 lb weight loss over the previous several months. The blood pressure was 165/102 mmHg, heart rate was 80, and he was afebrile. Physical exam was otherwise unremarkable with a clear chest, no peripheral lymphadenopathy, no rash, and no edema. Laboratories (Table 1) were remarkable for Ca 13.5 mg/dL, creatinine 7.6 mg/dL, and phosphorus 7.4 mg/dL. Urinalysis showed calcium-oxalate crystals with 4–10 RBCs/HPF with normal morphology and the urine albumin/creatinine ratio was normal at 24 mg/g. Evaluation of the hypercalcemia revealed the following: PTH < 3 (11–67 pg/mL), 25-hydroxyvitamin D 23.8 (30–95 ng/mL), 1,25-dihydroxyvitamin D 79 (18–72 pg/mL), and angiotensin converting enzyme (ACE) level 82 (9–67 U/L) (Table 2). Serum and urine immunofixations did not detect a monoclonal protein. A skeletal survey showed no lytic or blastic osseous lesions. Thyroid function tests were normal. His chest X-ray was negative and PFTs (pulmonary function tests) were normal but a computed tomography (CT) scan of the chest without contras showed mediastinal and hilar lymphadenopathy (Figure 1). An abdominal and pelvic CT showed a 3 mm nonobstructing left renal calculus with normal size kidneys and no nephrocalcinosis (Figure 3). Renal biopsy (Figure 2) showed granulomatous interstitial nephritis with diffuse interstitial inflammation with focal noncaseating granulomas. Acid-Fast Bacillus (AFB) and Grocott-Gomori's stain were negative for mycobacteria or fungal elements. Immunofluorescent microscopy demonstrated no significant staining for IgG, IgA, IgM, C3, C1q, kappa, lambda light chains, or fibrinogen. A diagnosis of sarcoid was made. The patient was initially treated with intravenous normal saline with improvement in serum calcium but no improvement in his serum creatinine. His calcium rebounded. There was suspicion for intrinsic renal disease as opposed to renal failure based on these findings. The patient was then started on prednisone 40 mg/day and the decision was made to obtain renal biopsy for definitive diagnosis. Once the renal biopsy results showed GIN, the patient was started on IV methylprednisone 60 mg/d for three days after which oral prednisone was continued at 1 mg/kg/day with slow taper planned over 12–18 months. With the addition of the higher dose of steroids his calcium normalized to 8.6 mg/dL and the creatinine decreased to 4.5 mg/dL on discharge. Six months after discharge his creatinine improved to 2.55 mg/dL and has remained stable with a normal serum calcium. With steroid treatment the 1,25-dihydroxyvitamin D level decreased to 19 (18–72 pg/mL) and ACE level normalized at 24 (9–67 U/L).

## 3. Discussion

Sarcoidosis is a systemic disease of unknown cause that is characterized by the formation of immune granulomas in various organs, mainly the lungs and the lymphatic system [2]. Sarcoidosis can involve any organ but in more than 90 percent of patients it manifests with pulmonary involvement. Respiratory symptoms include cough, shortness of breath, and chest discomfort [3]. Most patients with GIN due to sarcoidosis present with extrarenal manifestations such as pulmonary, skin, or eye involvement [4, 5]. However, there are a few series reporting sarcoid GIN without extrarenal involvement [6–8]. Our patient did not have any respiratory symptoms and had a normal chest X-ray in addition to normal PFTs. His chest CT showed asymptomatic mediastinal and hilar lymphadenopathy. Renal failure commonly ranges from 0.7% to 4.3% of cases in previous reported clinical series of patients with sarcoidosis but renal failure from GIN itself is rare [1, 9]. A previous study found that 46 of 9,779 (0.5%) renal biopsy specimens had GIN [10]. The pathology contributing to AKI from GIN in sarcoidosis is thought to be due to noncaseating granulomatous inflammation, which is composed of a central follicle of macrophages, epithelioid cells, and multinucleated giant cells [9, 11, 12].

Hypercalcemia, a well-known metabolic complication of sarcoidosis, is only found in 10–20 percent of patients and can directly cause acute kidney injury from renal vasoconstriction and volume depletion as a result of nephrogenic diabetes insipidus [13]. Hypercalcemia is due to overproduction of 1,25-dihydroxy vitamin D. The normal conversion of 25-hydroxyvitamin D to 1,25-dihydroxyvitamin D (calcitriol) occurs in the kidney through 1-*α* hydroxylase, a cytochrome p 450 enzyme [9, 14]. In sarcoidosis and other granulomatous diseases pulmonary macrophages express 1-*α* hydroxylase, which is often resistant to negative feedback mechanisms causing overproduction of l,25-(OH)2-D3 [9, 15] leading to increased calcium uptake by the gut. Adams et al. demonstrated that l,25-(OH)2-D3 is the hypercalcemia-causing factor in sarcoidosis and that macrophages from patients with sarcoidosis are the synthetic source of hormone in the disease. Mason et al. identified a similar metabolite in preparations of sarcoid granulomas incubated with 25-OH-D [11, 16, 17]. In patients with sarcoid, hypercalciuria is three times more common than hypercalcemia [11, 18] with a frequency in some studies as high as 60% [19]. Both can lead to acute and chronic kidney injury in sarcoidosis by causing nephrolithiasis and nephrocalcinosis. Hypercalcemia and hypercalciuria contribute to the formation of calcium oxalate crystals which was likely the cause of nephrolithiasis in our patient. Interstitial calcium oxalate deposition is also seen in association with granulomas in sarcoidosis [20].

The differential diagnosis of hypercalcemia was initially broad for our patient and included hyperparathyroidism, malignancy related (multiple myeloma, lymphoma, PTHrp associated malignancy, and metastatic bone disease), infections such as tuberculosis, sarcoid, and vitamin D intoxication. Laboratory assessment narrowed the differential with an appropriately suppressed PTH and a low 25-hydroxyvitamin D level. The negative serum and urine immunofixations and absence of lytic or blastic lesions on a skeletal survey made malignancy less likely. The elevated ACE and 1,25-dihydroxyvitamin D level made sarcoid a strong possibility but lymphomas can also cause increased production of 1,25-D. Intrinsic renal disease was higher on the differential rather than renal failure from nephrocalcinosis based on the following reasons: (1) while the hypercalcemia was slowly improving with intravenous hydration, the serum creatinine did not improve. (2) the CT of the abdomen and pelvis showed a 3 mm nonobstructing left renal calculus with normal size kidneys and no nephrocalcinosis (Figure 3). Moreover, the renal biopsy was required for definitive diagnosis. In sarcoidosis, with the exception of Löfgren's syndrome, all other suspected cases require a biopsy specimen to establish diagnosis from the involved organ that is most easily accessed [21]. In our patient, the involved organ was the kidney. Since the patient had renal symptoms and no pulmonary symptoms (PFTs were normal and there were no clinical pulmonary symptoms) or skin involvement, the decision was made to proceed with a renal biopsy. It was ultimately the renal biopsy which demonstrated GIN in the absence of another cause that led to a diagnosis and an effective treatment plan.

The primary treatment option for GIN due to sarcoidosis is glucocorticoid therapy. Renal limited sarcoidosis with GIN is a rare occurrence but several case reports suggest that these patients do well with corticosteroid treatment [22–24] although Ikeda et al. report a case of GIN due to sarcoidosis requiring dialysis [25]. Early diagnosis and treatment may be necessary to prevent progression. Robson et al. hypothesized that idiopathic granulomatous interstitial nephritis may actually represent a renal-limited form of sarcoid. It may be associated with hypercalcemia and an elevated serum angiotensin-converting enzyme and usually responds to treatment with corticosteroids. They describe a number of patients with biopsy proven GIN without extrarenal sarcoid who also presented with hypercalcemia and renal failure all of whom responded well to steroids [26]. Hilderson et al. present a detailed overview of current treatment options for renal sarcoid with hypercalcemia. They highlight the fact that treatment guidelines are lacking and that a uniform approach is needed in treating these patients. Variation exists between the treatment of hypercalcemia in sarcoidosis and GIN sarcoidosis. Hypercalcemia in sarcoidosis is initially treated with IV saline hydration followed by prednisone at a dose of 0.3–0.5 mg/kg once daily with a maintenance dose of 5–10 mg/day and the total duration of treatment being at least 12 months. However, for GIN sarcoidosis, they suggest three days of intravenous methylprednisolone followed by oral prednisone 1 mg/kg/d in patients with major organ impairment. The dose of steroids may vary depending on severity of disease with total duration of treatment being 18–24 months including a steroid taper. In cases of glucocorticoid failure or contraindications, immunosuppressive agents such as azathioprine or mycophenolate mofetil have been used. In cases of steroid resistant sarcoidosis and when at least one other immunosuppressive agent has been tried, TNF-alpha inhibitors have shown promise [27, 28]. Our patient was initially started on prednisone 40 mg per day while awaiting the results of the renal biopsy; once the biopsy results showed GIN the patient's treatment was tailored towards the diagnosis with significant improvement in overall condition. Thus, a renal biopsy should be performed when the suspicion for renal sarcoidosis is high without any other organ involvement in order to make a definitive diagnosis and guide management.

In conclusion, sarcoidosis is a disease involving multiple different organs including the kidney. Acute kidney injury as the initial presentation of sarcoidosis as was seen in our case is a rare entity. It is necessary to combine clinical presentation, laboratory results, and renal pathology to make a correct diagnosis which often responds well to treatment with steroids.

## Figures and Tables

**Table 1:** Lab values during hospitalization and after discharge.

Variable	Baseline labs, 6 months before admission	Hospital day 1	Hospital day 2	Hospital day 6	Hospital day 12,1 time dexamethasone was given	Hospital Day 16 (prednisone 60 mg Qd started)	Day 18 (discharge day)	15 days after discharge	Reference range
Sodium	139	135	134	136	138	138	135	139	136–146 mmol/L
Potassium	4.4	5.3	5.3	4.9	5.2	5.1	4.3	4.9	3.5–5.1 mmol/L
Chloride	104	101	106	112	104	110	111	104	96–107 mmol/L
Carbon dioxide	25	21	17	18	16	17	17	19	22–30 mmol/L
Blood urea nitrogen	23	57	57	40	57	65	67	68	8–24 mmol/dl
Creatinine	1.79	7.59	7.6	6.49	6.79	4.98	4.52	2.89	0.66–1.25 mg/dL
Glucose	137	167	148	99	106	216	145	94	74–106 mg/dL
eGFR	40	8	7	9	9	12	14	23	>90
Calcium	10.5	13.5	12.9	10.9	13.1	9.3	8.1	9.6	8.4–10.3 mg/dL
Corrected calcium^*∗*^	10.4	Unable to calculate	13.4	11.8	13.2	10.3	Unable to calculate	9.8	8.5–10.5 mg/dL
Ionized Ca	Not checked	Not checked	1.7	Not checked	Not checked	Not checked	1.12	Not checked	1.16–1.32 mmol/L
Phosphorus, inorganic	Not checked	7.4	6.1	4.7	7.5	Not checked	Not checked	3.4	2.5–4.5 mg/dL
Protein, total	7.3	Not checked	6	5.5	7.2	5.3	Not checked	6.2	6.3–8.2 g/dL
Albumin	4.1	Not checked	3.4	2.9	3.9	2.8	Not checked	3.7	3.5–5 g/dL
Bilirubin, total	0.6	Not checked	0.6	0.3	0.5	0.3	Not checked	0.4	0.2–1.3 mg/dL
Bilirubin, direct	0.1	Not checked	Not checked	Not checked	Not checked	0.2	Not checked	0.3	0.0–0.4 mg/dL
ALP	68	Not checked	43	42	113	66	Not checked	67	38–126 U/L
AST	26	Not checked	22	21	23	28	Not checked	20	15–46 U/L
ALT	24	Not checked	23	28	39	52	Not checked	25	13–69 U/L
WBC	12.8	12.8	13.2	8.6	10.8	18.7	13.1	14.8	3.4–11 k/*μ*L
Hemoglobin	13	13	11.2	10.6	12.1	10.3	9.9	12.4	13.0–17 g/dL
Hct	39.2	39.2	33.6	33.1	37.2	32.1	31.8	37	38–51%
Platelet	386	386	302	298	295	306	105	300	150–450 k/*μ*L
MCV	83.2	83.3	84.1	85.9	85.3	85.9	85.5	87.6	80–100 fL
Eosinophils (%)	5.2	5.2	3.9	6.7	7.1	0.4	Not checked	2.1	0.0–0.6%
Neutrophil (%)	77.6	77.6	77.8	70	69	91.1	Not checked	91	40–74%
Lymphocytes (%)	9	9	10	12.7	13.6	4.8	Not checked	4.3	18–44
Monocytes (%)	7.7	7.7	7.9	10.1	10.1	3.7	Not checked	2.4	4.7–12.0%
Basophil (%)	0.3	0.5	0.4	0.5	0.2	0	Not checked	0.2	0.1–1.4%

^*∗*^The normal albumin level is defaulted to 4.

**Table 2:** Lab values.

Variable	Measurement	Reference range
LDH	478	313–618 U/L
Creatinine kinase	40	55–170
Cholesterol, total	187	<200 mg/dL
HDL	28	>40 mg/dL
LDL	87	<130 mg/dL
Cholesterol/HDL ratio	6.7	0.0–4.9
Triglycerides	362	<151 mg/dL
ESR	26	1–13 mm/hr
CRP	Not checked	
ACE, before steroid treatment	82	9–67 U/L
ACE, after steroid treatment	24	9–67 U/L
Vit D, 25 hydroxy	27.8	30–95 bng/mL
Vit D, 1,25 hydroxy, before steroid treatment	79	18–72 pg/mL
Vit D, 1,25 hydroxy, after steroid treatment	19	18–72 pg/mL
PTH, intact	3.72	11–67 pg/mL
ANA	Negative	Negative
Immunofixation, serum	Polyclonal pattern	
IgG, serum	1330	700–1600 mg/dL
IgA, serum	187	70–400 mg/dL
IgM, serum	44	40–230 mg/dL
Immunofixatin elec., urine	Polyclonal IGG and polyclonal light chains	
Protein, random urine	10	mg/dL
C3	119	90–180 mg/dL
C4	22	10–40 mg/dL
Quantiferon-Tb gold	Indeterminate	
Mitogen-nil	0.16	IU/ML
NIL	0.03	IU/ML
TB Ag-nil	0	IU/ML
ASO Ab	46	>200 IU/ML
ANCA vasculitides		
Proteinase 3 Ab	<1.0	<1.0
Myeloperoxidase Ab	<1.0	<1.0
Hep A Ab, IgM	Nonreactive	Nonreactive
Hep A Ab, total	Reactive	Nonreactive
Hep B sAg	Negative	Negative
Hep B Core Ab, total	Reactive	Nonreactive
Hep BS ab	Reactive	Nonreactive
HepC Ab	Negative	Negative
HIV 1/2 Ab screen, rapid	Nonreactive	Nonreactive
HgbA1c	6.9	4.2–5.9%
Urine culture	Negative	Negative
Urine chemistry		
Protein, random urine	21	mg/dL
Microalbumin, random, urine	2.4	mg/dL
Sodium, random, urine	105	30–90 mmol/L
Potassium, random, urine	14.9	mmol/L
Calcium, random, urine	11.4	mg/dL
Thyroxine, free	0.61	0.8–1.5 ng/dL
TSH	1.038	0.4–4.2 *μ*IU/mL
PSA free	0.9	ng/mL
PSA percent free	43	>25%
Total PSA	2.1	≤4.0
